# Experimental Study of the Impact of Pore Structure on Drying Kinetics and Sublimation Front Patterns

**DOI:** 10.3390/pharmaceutics14081538

**Published:** 2022-07-23

**Authors:** Maximilian Thomik, Sebastian Gruber, Anders Kaestner, Petra Foerst, Evangelos Tsotsas, Nicole Vorhauer-Huget

**Affiliations:** 1 Department of Thermal Process Engineering, Institute of Process Engineering, Otto-von-Guericke University Magdeburg, Universitaetsplatz 2, 39106 Magdeburg, Germany; evangelos.tsotsas@ovgu.de (E.T.); nicole.vorhauer-huget@ovgu.de (N.V.-H.); 2 Department of Process Systems Engineering, TUM School of Life Sciences, Technical University of Munich, Gregor-Mendel-Str. 4, 85354 Freising, Germany; sebi.gruber@tum.de (S.G.); petra.foerst@tum.de (P.F.); 3 Laboratory for Neutron Scattering and Imaging, Paul Scherrer Institute, 5232 Villigen, Switzerland; anders.kaestner@psi.ch

**Keywords:** lyophilization, neutron imaging, in operando visualization, image processing, X-ray tomography, drying rates, ice saturation profiles

## Abstract

Freeze-drying frozen maltodextrin solutions with solid contents of 5% and 30% (*w/w*) was experimentally investigated using neutron imaging at PSI Villigen/Switzerland. Different solid contents, as well as annealing at −5 °C for 11 h, were used to modify the porous structure of the samples, which was quantified using X-ray computed tomography. Annealing of the 5% (*w/w*) sample, with a pore size distribution (PSD) of 23.7 ± 11.1 µm, yielded a very open pore space with high porosity (ε = 0.96) and a PSD of 33.0 ± 27.0 µm. In contrast, the higher solid content resulted in small, lamellar, narrow pores with high anisotropy and a porosity of ε = 0.65, as well as a PSD of 13.5 ± 4 µm. In operando neutron imaging was used to show the impact of the structure of frozen maltodextrin on the overall drying kinetics and shape of the sublimation front during freeze-drying. For this purpose, a freeze-drying stage was employed, which allowed a novel approach to time- and space-resolved monitoring of the ice phase. The sublimation front propagation was quantitatively analyzed based on ice saturation profiles and sublimation rates. The dependence of drying velocity on structure is nicely demonstrated by the data. In addition, it is shown that the sublimation front widened during freeze-drying, resulting in either rather concave or convex shape depending on morphological parameters.

## 1. Introduction

Drying is generally an energy intensive process. This is not only explained by the high phase change enthalpy of water alone, but also by the sluggish kinetics of heat and water vapor transfer. The limitation of transport kinetics generally results in long drying times and an increase in an already high energy consumption. In addition, freeze-drying, or lyophilization, is conducted at low temperatures and pressure, which further impairs drying kinetics [1,2,3,4] and increases energy consumption. Increasing the temperature in the freeze-dryer accelerates the process [5]; however, it also increases the risk of structural damage to the material [6,7]. Consequently, large safety margins are usually applied to guarantee a stable product quality. Better control of processing conditions is expected to be achieved if the dynamics of freeze-drying conditions can be improved [8]. One current approach is the correlation of heat and mass transfer kinetics with the porous structure of the material [9,10]. This is especially relevant for materials with a pore size distribution (PSD), i.e., where the ice and solid content are inhomogeneously distributed. In this case, local heat transfer varies in accordance with structure variation. In addition to that, varying pore sizes also affect vapor transport through dry pores; generally, larger pores impose lower mass transfer resistances than smaller ones. The heterogeneity of transport conditions resulting from distributed pore space can affect the development of the sublimation front, which propagates faster in larger pores. The result is a structured sublimation front, a phenomenon such as described in [11,12,13,14,15,16]. Hence, the freezing process, i.e., the development of the solid matrix, was investigated in depth [17,18,19] to evaluate and subsequently find pore structures favorable to heat and mass transfer.

The dynamic temporal evolution of the sublimation front can be assessed using in operando imaging techniques with high contrast between the propagating dry pores, solids and ice [12,13,15,16,20,21]. At the same time, pore scale models can assess heat and mass transfer kinetics at the front. Pore network models are a common tool to study drying processes in porous media [22,23], but have not yet been widely applied to study freeze-drying, except for our own work [24,25]. Such models can be built using realistic data for the porous material (e.g., data obtained using micro-computed X-ray tomography (µ-CT)); therefore, they can predict interesting structure–transport correlations.

In this work, we study the dependence of freeze-drying kinetics on pore structure based on the complete visualization of the sublimation front propagating inside frozen maltodextrin solutions, using in operando experiments with neutron imaging techniques and µ-CT [26,27]. While neutron imaging provides a good contrast between water-ice, solid maltodextrin and pores, it has a comparably low local resolution. For this reason, µ-CT, with a high resolution of up to 1 µm, is used to obtain additional necessary information regarding the pore size distribution of the dried samples.

The investigated maltodextrin samples were prepared with different solid contents and annealings to generate different porous morphologies. Freeze-drying was conducted at constant conditions using a freeze-drying stage that enabled in operando neutron imaging during the process. In addition to continuous radiographic imaging, tomography was also conducted to obtain more details regarding the structure of the sublimation front.

Radiographic images were used to determine freeze-drying kinetics in terms of the temporal evolution of the overall ice saturation, as well as sublimation fluxes. In addition, the sublimation front structure was qualitatively characterized. As a major outcome of the study, it can be shown that the solid content, as well as annealing, changed the pore structure significantly, which had a severe effect on the global freeze-drying kinetics. Additionally, different sublimation front structures were observed, depending on the structural properties of the maltodextrin samples, varying between roughly convex and concave shapes. In all cases studied here, the sublimation front recognizably widened without an apparent effect on the sublimation rates. These data serve as a base for future studies of the pore scale effects that occur during freeze-drying.

## 2. Experimental Materials and Methods

### 2.1. Experiments

#### 2.1.1. Experimental Equipment

**Figure 1:**
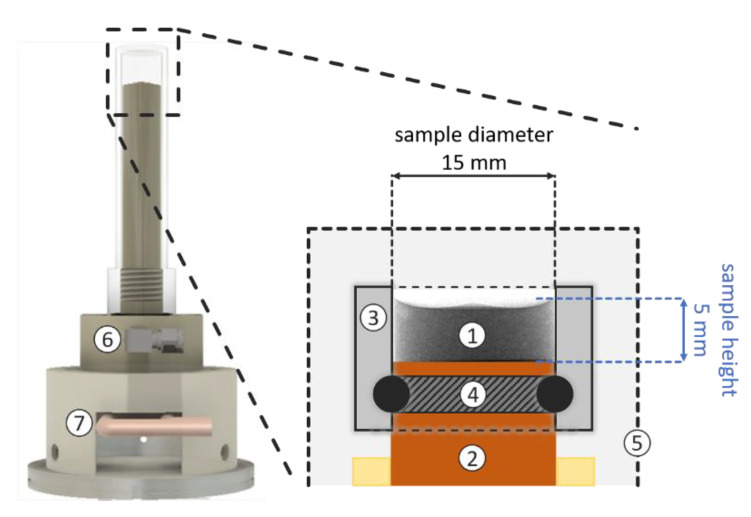
Freeze-drying stage with sample: (1) sample; (2) Peltier element inside cooled copper housing; (3) sample case; (4) rubber seal; (5) capsule; (6) vacuum connection and (7) Peltier element with cooling system (modified illustration from [28]).

All freeze-drying experiments were conducted inside the freeze-drying stage illustrated in Figure 1 [28]. This freeze-drying stage was designed to replicate the geometrical conditions in vials often used in commercial freeze-drying processes. The cylindrical sample case had an inner diameter of 15 mm (Figure 1). The sample case and the capsule were made of aluminum to provide good penetration properties for neutrons. Samples were cooled and heated using a Peltier element installed in the copper housing, which was in direct contact with the samples [29]. The Peltier element itself was cooled by an external cooling system based on ethylene glycol (30:70) set to −3 °C. Temperature inside the cell was measured using a thermocouple (PT1000) close to the sample position, i.e., near the shelf surface. The cell was evacuated by a vacuum pump (E2M1.5, Edwards, Feldkirchen, Germany) connected to a PTFE pipe with a 6 mm inner diameter. A magnet valve and Vacubrand controller unit (CVC3000 controller, VV6 valve) were used to control the evacuation and ventilation of the cell during drying and imaging. Pressure was measured using a Pirani sensor (VSP3000, Vacuubrand GmbH u. Co KG, Wertheim, Germany) located inside the exhaust pipe approximately 3 m away from the cell. This long distance was necessary to avoid activating the equipment with the neutron beam.

#### 2.1.2. Sample Preparation

Solutions of maltodextrin DE12 Glucidex from Roquette (Lestrem, France) were used in all experiments. Solutions were prepared with D_2_O (99.9% D, VWR chemicals, Radnor, PA, USA) to enable a better visualization when imaging the sublimation front [30]. Solutions were prepared with maltodextrin concentrations of *c* = 0.05 *w*/*w* and *c* = 0.3 *w/w* (Table 1). Three samples, each with volumes of 900 µL (fill depth of 5 mm), were prepared. They were cooled at 0.1 K/s in the freeze-drying stage (Figure 1). The target temperature for freezing was −15 °C in all cases, which is slightly above the glass transition temperature of maltodextrin DE12, which was estimated to be −12 °C using differential scanning calorimetry (DSC 214 Polyma, NETZSCH-Gerätebau GmbH, Selb, Germany). The temperature was held for 30 min at −15 °C to ensure homogeneous and stable conditions. One sample (*c* = 0.05 *w/w)* was then annealed for 11 h at −5 °C and subsequently cooled again at −15 °C using the same cooling rate.

#### 2.1.3. Experimental Procedure and Parameters

All freeze-drying experiments were carried out in identical process conditions (*T* = −15 °C and *P* = 10 Pa). To start the freeze-drying process, the pressure was reduced to the target pressure of 10 Pa at a constant temperature of −15 °C. During the experiments, radiographic images were continuously taken. Tomographies were recorded at distinct drying times. For the tomography scans, the freeze-drying stage was vented with air to pressurize the setup and interrupt drying. At the end of drying the temperature was slowly raised to ambient temperature at a rate of 0.01 K/s, and vented to atmospheric pressure to stabilize the sample. Samples were safely removed from the freeze-drying stage and stored in a container until they were required for µ-CT imaging. 

#### 2.1.4. Neutron Facility and Imaging Parameters

Neutron imaging of the freeze-drying experiments was carried out at the cold neutron instrument, ICON [31]. ICON is located at the SINQ neutron source of the Paul Scherrer Institute in Villigen, Switzerland. Detection was realized using the micro setup [32] and an Andor ikon-L camera featuring a 2048 × 2048 pixel chip. The scintillator was a 30-µm-thick Gadox + 6LiF:ZnS screen (from RCTRITEC, Teufen, Switzerland). The exposure time per image was 15 s. At the measuring position, an L/D ratio of 343 was yielded [33]. The field of view was 27 × 27 mm^2^ and the voxel size was 13.5 µm. A voxel is the smallest 3D cubical element in an image, limited by the given size. The dimension of the region of interest (ROI) (Figure 1) was calculated from the number of pixels and the pixel size; it is 4.86 mm (360 pixels) in height and 15.4 mm (1140 pixels) in diameter. The small height is a result of cropping the image at the upper side of the sample, where the frozen sample had an undefined structure resulting from filling and freezing.

The initial intensity ($I_{0}$) of the neutron beam was attenuated during transmission through the material between the source and the detecting unit. The transmitted intensity (I(t)) was measured using the detecting unit and translated into a gray value image. Attenuation was influenced by the linear attenuation coefficient ($\mu_{i}$) and thickness ($z_{i}$), as well as the composition of the material, according to the Lambert–Beer law

(1) $$I\left(t\right)=I_{0}\cdot e^{-\sum \mu_{i}z_{i}}$$

A change in composition due to the sublimation of ice during the process resulted in a change of detected intensity (I(t)) and, thus, varying gray values, as follows

(2) $$\sum \mu_{i}z_{i}=\mu_{stage}z_{stage}+\mu_{D_{2}O}z_{D_{2}O}+\mu_{malto}z_{malto}$$

The low attenuation of aluminum ($\mu_{stage}$ = 0.042 cm^−1^) and the low density of maltodextrin ($\mu_{malto}$ = 4.9 cm^−1^) yielded a reasonable transparency for neutrons. In contrast, *D*_2_*O* (${µ}_{D_{2}O}=0.587\;{\mathrm{cm}}^{-1})\;$absorbed most of the radiation, which enabled very good contrast between saturated and unsaturated regions of the samples, clearly forming the base for investigation of sublimation front evolution during freeze-drying.

#### 2.1.5. Analysis of Pore Structure after Freeze-Drying

Samples dried inside the neutron facility were afterwards investigated using µ-CT (XCT-1600 HR system from Nordson Matrix Technologies GmbH, Feldkirchen, Germany) (Table 2). Reference samples, dried outside the neutron facility under identical conditions, were additionally investigated using µ-CT with the parameters given in Table 3. Samples from the neutron imaging experiment were scanned completely, with a voxel size much greater than the minimum pore size, i.e., 8 µm (Table 2). The PSDs were instead determined based on µ-CT imaging of samples obtained from identical freeze-drying experiments conducted outside the neutron facility. The samples were cut into smaller pieces to allow for maximum resolution (voxel size 1 µm). The reconstructed volumes had an even smaller size than these subsamples, namely 400 × 400 × 400 voxels. These domains were extracted from different regions of the subsamples with homogeneous pore structure; thus, they represented only a small part of the overall sample.

Multiple radiographs (Table 2 and Table 3) were taken for each projection during each scan. The average of the radiographs was used to reconstruct a 3D volumetric image. This approach generally results in higher quality images and reduced noise. The scans were subsequently reconstructed as a 3D volumetric image with 16-bit encoding using custom-designed software, MIPS-CT v5.1.6.0 (Nordson Matrix Technologies), that uses CERA v5.1.0 (Siemens Healthcare GmbH, Erlangen, Germany) for visualization. Images were binarized for analysis of the pore structure. Lower concentration samples were binarized via Otsu thresholding after a median filter was applied; the high concentration sample was binarized via adaptive thresholding after an anisotropic diffusion filter was applied. Pore space was segmented into individual pores using watershed segmentation. As described in [25], pore diameters of all samples were calculated according to the equivalent diameter method. For the MD30 method with lamellar pores, the hydraulic diameter is a suitable alternative approach [17].

### 2.2. Image Processing

First, each radiographic image of the neutron imaging dataset containing information regarding the detected intensity, I(t), was corrected utilizing the following methods:

1. *Gamma Spot* correction to remove unwanted white “gamma spots” from images [31];

2. *Dark Field* (*DF*) correction to eliminate grayscale deviation caused by the equipment (e.g., the camera); 

3. *Open Beam* (*OB*) correction to remove background noise.

From these steps, the corrected images, T(t),

(3) $$T\left(t\right)=-ln\left(\frac{I\left(t\right)-DF}{OB-DF}\right)$$

were obtained. Using the dry reference image taken at the end of freeze-drying, it was possible to quantify temporal and local changes in the ice saturation, S(t), of partly dried samples:

(4) $$S\left(t\right)=\frac{\bar{T}\left(t\right)-\bar{T}\left(t_{end}\right)}{\bar{T}\left(t=0\right)-\bar{T}\left(t_{end}\right)}$$

Global saturation, S(t), was calculated based on the mean gray value of an image at a given time, $\bar{T}\left(t\right)$; the gray value of the dry image, $\bar{T}\left(t_{end}\right)$; and the completely saturated image from the start of drying, $\bar{T}\left(t=0\right)$. The water content was then calculated from:

(5) $$M_{D_{2}O}\left(t\right)=S(t)\cdot M_{D_{2}O}\left(t=0\right)$$

using the values for $M_{D_{2}O}\left(t=0\right)$ from Table 1. The sublimation rate, ${\dot{M}}_{sub}$, was calculated from the change in saturation between two consecutive images:

(6) $${\dot{M}}_{sub}=\frac{S(t)-S(t_{-1})}{t-t_{-1}}\cdot M_{D_{2}O}\left(t=0\right)$$

The sublimation flux, ${\dot{m}}_{sub}$ from the sample surface, with approximately $A_{surf}$ = 177 mm^2^, finally resulted from:

(7) $${\dot{m}}_{sub}=\frac{{\dot{M}}_{sub}}{A_{surf}}$$

## 3. Results

### 3.1. Porosity and PSDs

**Figure 2:**
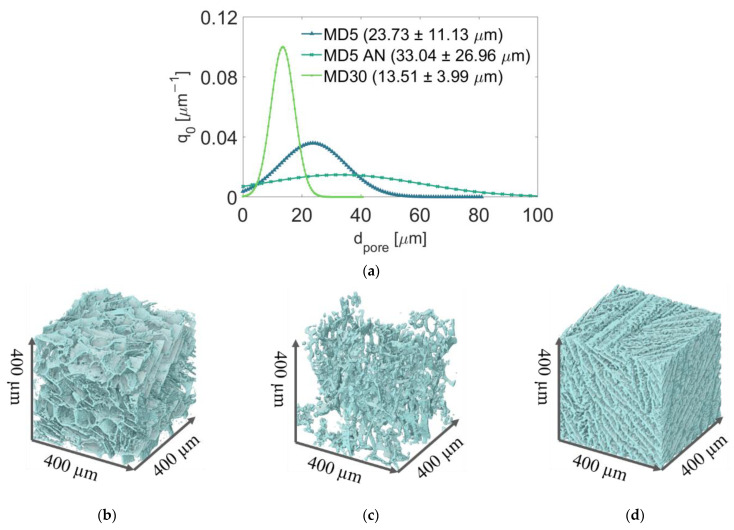
(**a**) Average pore size distribution of each of three samples extracted from different positions; 3D renders of representative parts of samples: (**b**) MD5, (**c**) MD5 AN and (**d**) MD30.

The average PSDs for each sample are shown in Figure 2a. Visual representations of reconstructed µ-CT images are provided in Figure 2b-d. The comparison reveals monomodal PSDs, confirming homogeneity of the small scanned porous domains. Additionally, Figure 2 shows that the higher solid content of MD30 yielded overall smaller pore sizes and a narrower PSD. In contrast to MD5 and MD5 AN, MD30’s solid structure can be characterized as lamellar, resulting in long and narrow pores. Furthermore, the pore structure appears anisotropic, i.e., with pores oriented in a preferential direction. This is a result of the freezing process [18,19].

Annealing increased pore sizes and widened PSD, with a significant increase in porosity. This was expected based on data from the literature [12,34,35]. The MD30 sample had the smallest mean pore size (13.51 ± 3.99 µm); the mean pore diameter of MD5 was 23.73 ± 11.13 µm. Annealing resulted in a mean value of 33.04 ± 26.96 µm. The corresponding porosity values are ε_MD30_ = 0.65, ε_MD5AN_ = 0.96 and ε_MD5_ = 0.87, respectively. Thus, widening the pores towards larger values during annealing seemed to increase porosity. This is explained using the µ-CT resolution; pores smaller than 1 µm were not resolved in the case of MD5. The increase in pore size as a result of annealing allowed the detection of these pore volumes in the MD5 AN sample.

**Figure 3:**
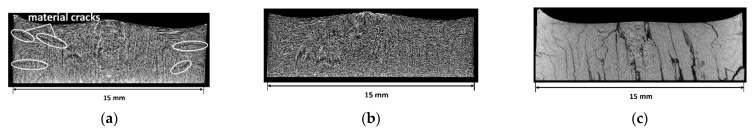
µ-CT imaging of the complete samples from in operando freeze-drying, with voxel sizes of 8 µm: (**a**) MD5, (**b**) M5 AN and (**c**) MD30. The images in (**a**) and (**c**) clearly reveal cracks propagating through the samples.

The small sample sizes used to obtain the data in Figure 2 basically do not allow detection of macroscopic heterogeneity; compromises to the domain size were made to allow a high spatial resolution. Such data are only accessible if µ-CT imaging is conducted for the whole sample, i.e., by accepting losses in the achievable resolution. In order to gain a broader view of the pore structure, such scans were conducted for all samples used in the neutron imaging experiment after drying. The results are shown in Figure 3. The achieved voxel size in these images is 8 µm. The images clearly reveal cracks propagating through the samples which were not treated by annealing (MD5, MD30). These cracks significantly change the macroscopic pore structure as they increase the local pore size. When these cracks developed is unclear, as µ-CT imaging was conducted after freeze-drying. As can be seen from the images in Figure 3a,c, these cracks have a vertical orientation in the center of the samples and a rather horizontal orientation along the sides of the samples.

### 3.2. Temperature and Pressure

**Figure 4:**
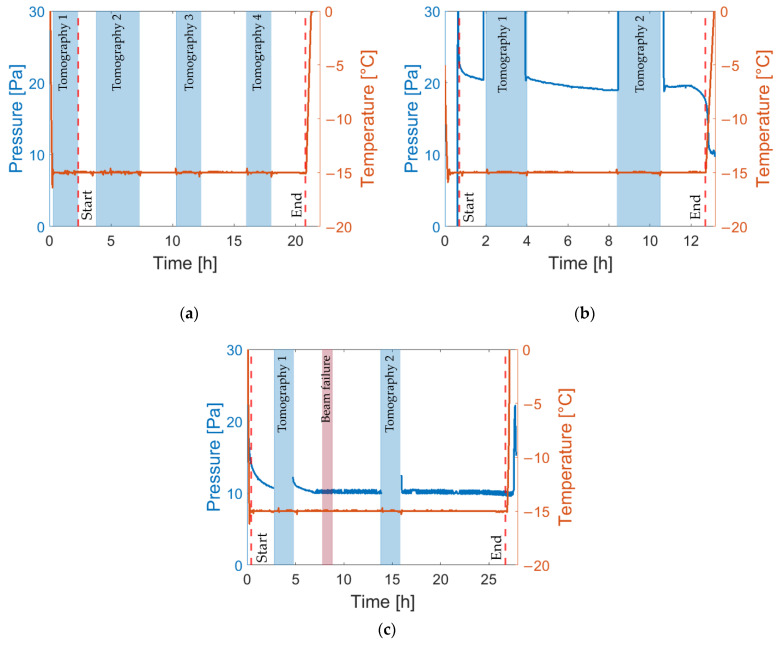
Pressure (blue lines) and temperature (red lines) profiles of (**a**) MD5, (**b**) MD5 AN and (**c**) MD30. The vertical dashed red lines indicate start and end of freeze-drying.

Figure 4 displays the measured temperature and pressure profiles of all conducted experiments. It is noted that the pressure of experiment 1 (freeze-drying of MD5) could not be recorded accurately, wherefore it is not shown in Figure 4a. In experiment 3 (freeze-drying of MD30), the neutron beam supply was interrupted, resulting in an interruption of imaging of the process. This short period is identified by “Beam failure” in Figure 4c. The segments at which tomographic images were captured are highlighted in blue. The start and end points of the freeze-drying processes are denoted by “Start” and “End”, respectively, in these diagrams.

As can be seen, the shelf temperature was kept constant during all experiments. The pressure of the system, measured using a Pirani sensor inside the exhaust pipeline, was decreased by the vacuum pump at the start of freeze-drying experiments, and increased by interrupting the pump for the purpose of tomography imaging, for which the freeze-drying processes were interrupted. The target pressure of *P* = 10 Pa could only be achieved in the case of MD30; in MD5 AN, the pressure remained at approximately 20 Pa until the end of drying. This can be explained by the higher water content and the higher sublimation rate in this experiment, as will be shown in the discussion that follows. Apart from that, the pressure shows an overall slightly decreasing trend, which is related to a reduction in the sublimation rate with increased drying time [36]. It is expected that the uncontrolled pressure only slightly affected drying time, but that the pressure was not high enough to result in partial material collapse [37].

### 3.3. Qualitative Comparison of Image Data

**Figure 5:**
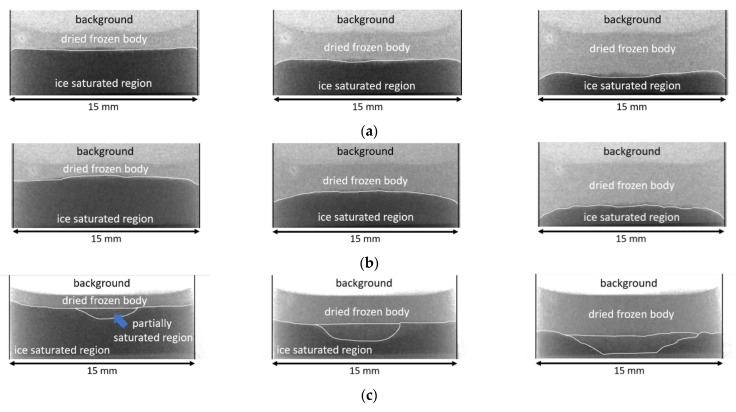
Evidence of structured sublimation fronts from radiographic images taken at ice saturations of S = 0.25, 0.5 and 0.75: (**a**) MD5, (**b**) MD5 AN and (**c**) MD30. In partially saturated regions the overlap of the dried and ice-filled regions results in saturation values between 1 and 0.

Selected image data from radiographies are summarized in Figure 5, after reaching approximately S = 0.75, 0.5 and 0.25 sample saturation. In these images, regions fully saturated with water appear dark gray and are identified as “ice saturated region”. The fully dried regions appear light gray and are identified as “dried frozen body”. The dried regions are darker when more material is accumulated and absorbing neutron radiation; i.e. for MD30 a darker dry region is observed than for MD5. A sublimation front formed between the two regions, and is highlighted in these images. It is characterized by a partially saturated section with different extensions in each of the three samples. In a 2D perspective, the ice-filled and already dried regions around the sublimation front overlap, creating partially dried regions in the images. In a 3D view, the front widens in vertical direction, resulting in the co-existence of water-ice filled and empty regions.

The partially saturated region develops because of the nonplanarity of the sublimation front; i.e., the most advanced point moves faster than the least advanced point. This phenomenon is much stronger in the case of MD30; for this reason, it is denoted as a “partially saturated region” in Figure 5.

It is clearly visible that the sublimation front’s shape differs in each experiment. Supposing similar and constant drying conditions based on data provided in Figure 4, it can be anticipated that the composition of the samples, as well as annealing, influenced the shape of the drying front. Such a relationship was already observed in other studies [21,38,39]. In our study, distinct sublimation front structures can be documented. More precisely, a flat sublimation front is observed in the experiment with low mass fraction of maltodextrin (MD5). In contrast, inside the annealed sample the sublimation front tended to form a convex shape (MD5 AN). In the sample with high maltodextrin content, a valley occurred in the center of the sample (MD30). It formed very early, i.e., when the sublimation front was still close to the open top surface. It became larger when the sublimation front travelled downwards through the sample. Finally, it almost spanned the whole cross-section at the end of drying.

**Figure 6:**
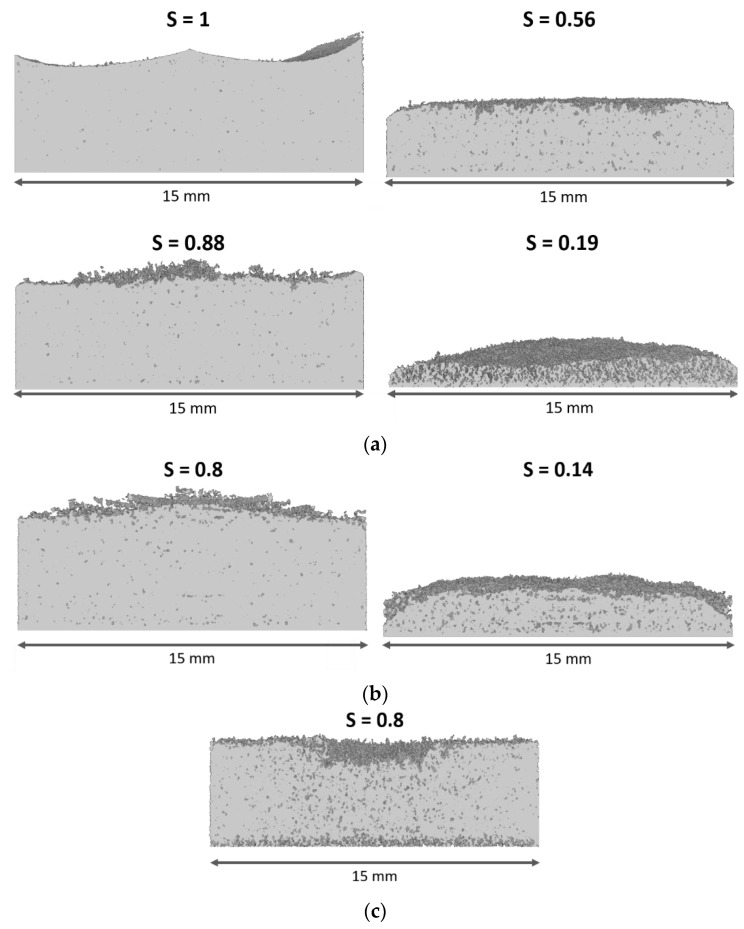
Neutron tomography imaging-based 3D visualization of samples at the specified saturations, S: (**a**) MD5, (**b**) MD5 AN and (**c**) MD30. Ice-saturated regions are presented in light gray, and partially dried regions in dark dray. Completely dry regions are not shown. Dark gray spots indicate the development of dry regions below the main sublimation front.

Tomography images are shown in Figure 6; note that the second tomography of MD30 failed and is not provided. Figure 6 shows that all samples had a similar shape at the start of freeze-drying. In the case of MD5 and MD5 AN, the front remained flat with slight progress along the edges, as already visible in Figure 5. The last tomography of MD5 also indicates that the drying front widened at the end of drying. This can be recognized by the dark gray regions, which represent dry parts of the sample; they are spread over the remaining ice-saturated zone, at S = 0.19, in Figure 6a. A similar behavior is observed in the MD5 AN sample (S = 0.14 in Figure 6b). Figure 6a,b also illustrate that ice clusters remained behind the main sublimation front. These clusters had a lower sublimation rate and dried when sublimation proceeded further downwards.

For MD30, a very different behavior is observed. As already shown in Figure 5c, a dry valley expands in vertical and horizontal directions in the center of the sample until the end of drying. The sample dries more heterogeneously, again identified by dry dark gray spots spread over the ice-saturated region, and seemingly also from the bottom side. This finding is similar to the experiment reported in [15], where maltodextrin with 20% (*w/w*) solid content was investigated using neutron imaging. There, distinct dry fingers developed in the center of the sample and resulted in faster drying of this region, whereas the ice ring, located around the center, dried more slowly.

Furthermore, the in-operando obtained tomography images of the samples do not reveal cracks at the given resolution of 13.5 µm. Since the cracks have a size (width) of up to 390 µm (MD30), an explanation might be their development during secondary freeze drying or their enlargement (above 13.5 µm) after primary freeze drying. 

In summary, very different drying behaviors were observed for different solid contents, characterized by different pore structures (Figure 2). While for MD5 and MD5 AN the front remained almost flat, in the sample with high solid content, a completely different shape was formed, with significant front widening, in agreement with previous findings [15].

### 3.4. Saturation Profiles and Sublimation Rate

**Figure 7:**
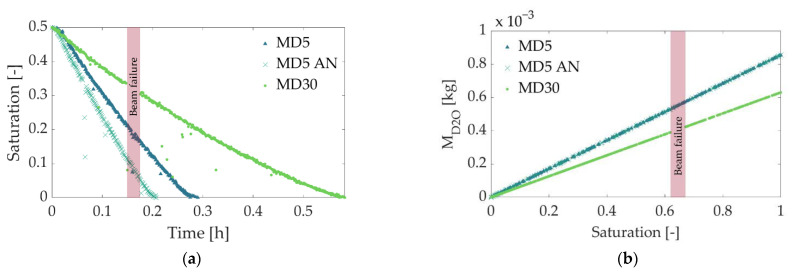
(**a**) Overall saturation over time and (**b**) corresponding water content. The beam failure occurred in experiment 3 (MD30) and is marked by the red bar.

The profiles of overall ice saturation vs. time were computed from the radiographic images (Equation (4)), which were partially presented in Figure 5. They are shown in Figure 7a, and useful for a quantitative comparison of the three experiments, as well as for the computation of sublimation rates. The corresponding water contents in Figure 7b were computed using Equation (5). Please note that failures of the neutron beam influenced the gray value in the images and partly caused spurious saturation values (outliers in Figure 7).

**Figure 8:**
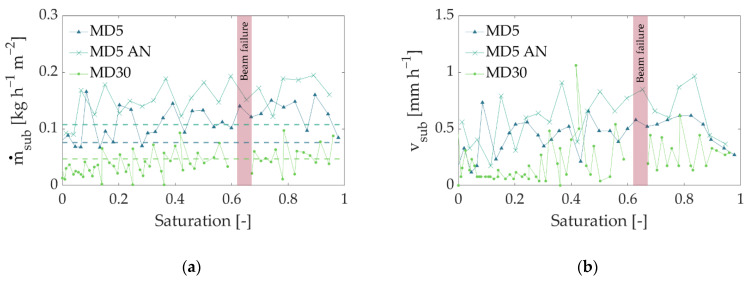
(**a**) Comparison of sublimation fluxes. The beam failure occurred in experiment 3 (MD30) and is marked by the red bar. (**b**) Velocity of sublimation fronts, calculated from the mean front positions.

The profiles clearly indicate three major aspects. First, the overall ice saturation decreased almost linearly over the longest drying period in all cases, but with different slopes, resulting in different drying times, depending on the mass fraction of maltodextrin and treatment by annealing. Finding linear decreases in saturation in all samples was surprising. In most cases, a nonlinear time dependency of the ice saturation was reported in the literature [4,40], clearly resulting from the receding of the sublimation front into the bulk material. Only a few studies document a linear trend similar to that observed in our experiments; however, in these examples, the temperature was continuously increased during the process [41,42] or heat was additionally supplied by microwaves [11]. In our experiment, the temperature was kept constant; therefore, it cannot explain the constant slope of the saturation profiles. Instead, other mechanisms must be considered here. One explanation might be vapor pressure inside the freeze-drying chamber. As shown in Figure 4, vapor pressure decreased with drying time; however, the measurement was realized at quite a long distance away from the sample (approx. 3 m) to avoid radioactive activation of the equipment. The pressure inside the cell might have been much higher, especially at the start of drying. In previous studies [21], it was found that the small opening of the drying chamber limits vapor transport from the vial to the vacuum pump, resulting in slow drying. High initial pressures could have decelerated drying in our experiment, whereas a reduction in vapor pressure over time might have had a superimposing positive effect on the sublimation rates (Figure 8).

As expected, the longest drying time was observed in the case of MD30, although its overall water content was lower than in the other two cases (Table 1 and Figure 7b). At the same time, the annealed MD5 sample (MD5 AN) revealed a significantly shorter drying time than the nonannealed sample with the same maltodextrin content (MD5). Similar observations were mentioned in [21], where they were explained by significant differences in pore structures. This finding is also in very good agreement with the literature [35,38], where a change in pore structure during annealing changed drying behavior.

Sublimation fluxes were calculated from the saturation profiles in Figure 7 using Equations (6) and (7). The linear saturation profiles result in almost constant sublimation fluxes (Figure 8) over the longest drying period. As expected, sublimation flux is high for the annealed MD5 sample (MD5 AN) and low for MD30. It is almost 1.5 times greater than that of the nonannealed MD5 sample, resulting in a reduction in drying time of approximately 3 h. Based on the mean sublimation front positions of all samples, $z_{fr}$ (i.e., the mean between the least and most advanced points of the fronts, determined using the local change in gray values in Figure 5), and the velocity of the sublimation front, $v_{sub}$ (Figure 8b), the sublimation flux can also be calculated from:

(8) $$v_{sub}=\frac{dz_{fr}}{dt}$$

or

(9) $${\dot{m}}_{sub}=\rho_{ice}\frac{\Delta z_{fr}}{\Delta t}$$

where $\rho_{ice}$ = 918 kg m^−3^ is the density of water-ice [43]. Equation (8) was solved for the average front velocity from Figure 8b; the result is shown in Figure 8a as dashed lines. As can be seen, the sublimation flux obtained from Equations (4)–(7) (based on the overall gray value saturation and the mass of ice contained in the sample) agrees very well with the solution using Equation (9).

However, a correlation between drying front structures and sublimation rates is not achieved, as microscale details are not captured by macroscopic variables. This can only be investigated based on a more detailed analysis of local processes, e.g., using pore network modeling.

### 3.5. Influence of Pore Structure

The PSDs from Figure 2 and porosity values correlate very well with observations in Figure 7 and Figure 8; MD5, with the largest porosity, had the highest sublimation fluxes and shortest drying time, and MD30, with small pores, had the lowest sublimation fluxes and the longest drying time. Considering freeze-drying in a vapor transport-controlled regime, the difference in pore sizes can explain the different freeze-drying rates in Figure 8 and drying times in Figure 7a. The narrow lamellar pores in the MD30 sample principally induce higher vapor transport resistances, hindering vapor transfer and reducing the overall sublimation rate. In addition, the anisotropic pore structure might have further hindered vapor transport. The large pores in the annealed sample yielded the opposite effect, i.e., higher sublimation rates. At the same time, the larger ice content in both MD5 samples might have positively affected heat transfer through the sample (thermal conductivity of ice: 2.33 W m^−1^ K^−1^; thermal conductivity of maltodextrin: 0.2 W m^−1^ K^−1^), again resulting in improved drying kinetics.

## 4. Summary and Conclusions

In this study, we investigated the dependence of drying kinetics on the structure of maltodextrin solutions frozen inside cylindrical drying chambers with a geometry similar to vials used in the pharmaceutical industry. The pore structures of the samples were determined using micro-computed tomography (µ-CT); the drying kinetics were obtained using neutron radiographic imaging, which allowed the exact determination of the time- and space-resolved ice saturation profiles. Pore size distribution (PSD) and porosity both varied with the solid content (5% and 30% (*w/w*)); additionally, annealing significantly widened PSD. At the same time, the development of cracks was reduced by the annealing treatment.

Measured sublimation fluxes were almost constant during the whole freeze-drying experiment, which might have been a result of vapor transport limitations inside the freeze-drying cell [16]. Only at the end of drying, at a saturation below *S* = 0.2, did the drying flux decrease slightly in the case of MD5 and MD5 AN. At the same time, the front widened as significant dry sections became apparent below the main sublimation front. This was clearly visualized by tomography images, which had a voxel size of 13.5 µm^3^. Apart from that, the samples with overall larger pores and higher porosity (5% (*w/w*)) revealed a higher sublimation flux and shorter drying time, although their initial water content was higher.

In addition to global drying kinetics, in operando neutron imaging allowed the time- and space-resolved detection of the sublimation front; the development of distinct front structures was apparent. While samples with a low mass content of maltodextrin formed flat-to-convex front structures independent of annealing, a very different behavior was observed for the sample with a high solid content (30% (*w/w*)). In this case, similar to earlier experiments reported in [15], the center region of the sample dried much faster than the rest of the sample, resulting in the formation of a distinct valley of dry and partially ice-saturated material. The distinct shape of the front was not reflected by the overall drying kinetics, which showed almost constant sublimation fluxes over the longest drying period, similar to the case of 5% (*w/w*). In previous studies, locally high sublimation rates were explained by local pore structure variation, mainly by larger pores located in the center of the sample. So far this is not justified by the data, as the µ-CT imaging structural analysis was either limited to small homogeneous subdomains, not considering the center section, or scans that did not exactly resolve the local structure. The latter, however, revealed large vertical cracks in the center of the 30% (*w/w*) sample, as well as in the nonannealed MD5 sample. However, as the scans were performed after the process finished, it is not yet clear exactly how and when these cracks developed. To clarify this question, the pore space would have to be already resolved during neutron imaging. Alternatively, pore scale modeling can help analyze and complete the data.

## Figures and Tables

**Table 1:** Sample preparation and preprocessing parameters.

Name of Sample	Annealing	Freezing Conditions (K/s)	Solid Content (*w/w*)	Sample Volume (µL)	D_2_O Content (g)
MD5	-	0.1	0.05	900	0.855
MD5 AN	11 h at −5 °C	0.1	0.05	900	0.855
MD30	-	0.1	0.3	900	0.630

**Table 2:** X-ray tomography imaging parameters for samples dried inside the neutron facility.

Parameters	Unit	MD5	MD5 AN	MD30
Acceleration voltage	(kV)	60	60	60
Current	(µA)	60	60	100
Exposure time	(ms)	1649	1434	984
Projections/360°	(-)	2000	2000	2000
Number of radiographs per projection	(-)	5	5	5
Voxel size	(µm)	8	8	8

**Table 3:** X-ray tomography imaging parameters for reference samples dried using the same parameters outside the neutron facility.

Parameters	Unit	MD5	MD5 AN	MD30
Acceleration voltage	(kV)	60	60	60
Current	(µA)	20	20	20
Exposure time	(ms)	5051	4776	4758
Projections/360°	(-)	2000	2000	2000
Number of radiographs per projection	(-)	3	5	4
Voxel size	(µm)	1	1	1
